# *Clostridium septicum* causing aortic aneurysm, spondylodiscitis, and epidural abscess in an immunocompetent man

**DOI:** 10.1186/s12879-025-11852-z

**Published:** 2025-11-29

**Authors:** Vindar Fritzell, Richard Dwyer, David Lindström

**Affiliations:** 1 Department of Infectious Diseases, Södersjukhuset, Sjukhusbacken 10, Stockholm, 118 83 Sweden; 2 Department of Clinical Science and Education, Vascular Surgery Section, Karolinska Institutet, Södersjukhuset, Sjukhusbacken 19, Stockholm, 118 83 Sweden

**Keywords:** Clostridium septicum, Aortic aneurysm, Epidural abscess, Spondylodiscitis

## Abstract

**Background:**

*Clostridium septicum* is an opportunistic pathogen residing in the human gut flora. Due to its aerotolerance and production of exotoxins, dissemination is an uncommon but potentially lethal condition, often associated with underlying malignancy. This report represents the first described case of a patient with aortic, musculoskeletal and epidural infection caused by *C. septicum.* It illustrates an example of successful treatment and discusses specific treatment options in relation to current antibiotic and surgical recommendations.

**Case presentation:**

We report a rare case of a 76-year-old man presenting with mild symptoms, who developed life-threatening complications caused by *C. septicum* including an aortic aneurysm as well as an epidural abscess and spondylodiscitis. The patient was successfully treated with endovascular stents in conjunction with a six-week course of intravenous antibiotics and was discharged from the hospital with oral clindamycin. After more than a year on oral antibiotics the treatment was terminated, which promptly led to a relapse of *C. septicum* bacteremia and spondylodiscitis. Following a second hospitalization with IV therapy, lifelong suppressive antibiotic therapy was deemed necessary.

**Conclusion:**

*C. septicum* bacteremia is a severe condition that may cause multifocal infection although presenting with few clinical signs. This unique case reflects the aggressiveness of the bacteria and how antibiotic and surgical treatment should be thoughtfully considered in the presence of aortitis, epidural abscesses and spondylodiscitis.

## Background

*Clostridium septicum* is a gram positive, obligate anaerobic, motile bacillus residing in the human gut flora. It is a spore-forming opportunistic pathogen that produces exotoxins such as the alpha toxin, which may cause intravascular hemolysis and gas gangrene leading to soft tissue necrosis [1, 2]. By translocating through ulcerations in the bowel mucosa, it can spread either hematogenously or directly in the peritoneal cavity, giving rise to rapidly progressing complications. *C. septicum* is more aerotolerant than other pathogenic clostridium species such as *C. perfringens*, which explains its ability to become seeded and cause potentially fatal septic foci [1, 2]. Moreover, *C. septicum* is associated with malignancy, colorectal and hematologic neoplasms [3].

*C. septicum* aortitis is a rare condition that emerges due to the bacterial invasion and inflammation of the vascular wall. Through the ability to produce spores and gas inside the wall, bacterial seeding of the vascular wall can lead to the development of infective native aortic aneurysms (previously called mycotic aneurysms) which are highly lethal. Vessels with atheromatous lesions may be more susceptible to C. septicum due to its hypoxic environment [4, 5]. Spontaneous hematogenous dissemination to the musculoskeletal system is also described, however only single events involving the spine have been reported [6–8].

## Case presentation

**Fig. 1:**
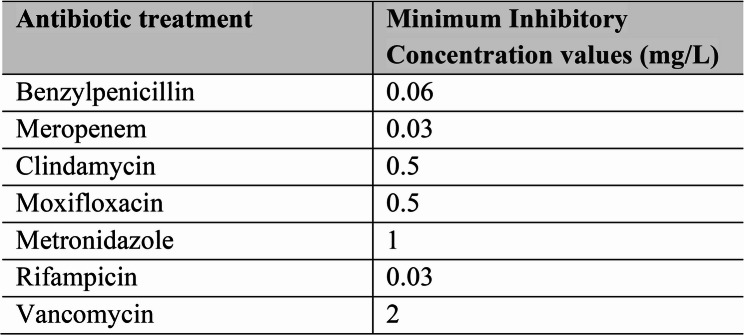
Table of all MIC values (mg/L) determined for *Clostridium septicum* in the anaerobic blood culture

A 76-year-old physically active man with type-2 diabetes and hypertension presented to the emergency department with fever of 40 °C and radiating lower back pain that developed on the same day, without any trauma prior to the onset of symptoms. Upon arrival, the patient had an oxygen saturation of 87% but was haemodynamically stable. Neurological examination was normal. On palpation of the spine, pain was triggered at the level of lumbar vertebrae (L) 2–3. Laboratory findings showed leukocytosis and a C-reactive protein (CRP) of 373 mg/L. The patient initially received one day of intravenous (IV) cefotaxime (2 g three times a day) empirically (Fig. 1.) followed by two days of IV piperacillin and tazobactam (4 g three times a day).

On day 3 of hospitalization, *Clostridium septicum* was identified in 4 out of 4 blood cultures (2 anaerobic and 2 aerobic bottles). The blood cultures were incubated in the BacT/Alert VIRTUO system (BioMérieux, Marcy-l’Etoile, France) at 36 °C until detection at 9.6 h (anaerobic bottle) and 82.6 h (aerobic bottle). The positive blood cultures were Gram stained on agar plates showing Gram positive rods. *Clostridium septicum* was identified using matrix assisted laser desorption ionization (MALDI-TOF) (Bruker, Massachusetts, USA). The analyses were carried out at the laboratory at the Division of Clinical Microbiology, Karolinska University Hospital (Stockholm, Sweden). MIC values were determined using the gradient test ETEST (BioMérieux, Marcy-l’Etoile, France), using the blood culture with the shortest time-to-detection, meaning the anaerobic blood culture in this case. As no species-specific breakpoints existed specific to *Clostridium septicum*, the Minimum inhibitory concentration (MIC) values were interpreted using the EUCAST guidelines on anaerobic bacteria [9].

Computed tomography (CT) and magnetic resonance imaging (MRI) of the back revealed spondylodiscitis of the L2-L3 intervertebral disc with surrounding gas accumulation and edema involving the right psoas muscle and erector spina, as well as an epidural phlegmon extending from L1-L4.

**Fig. 2:**
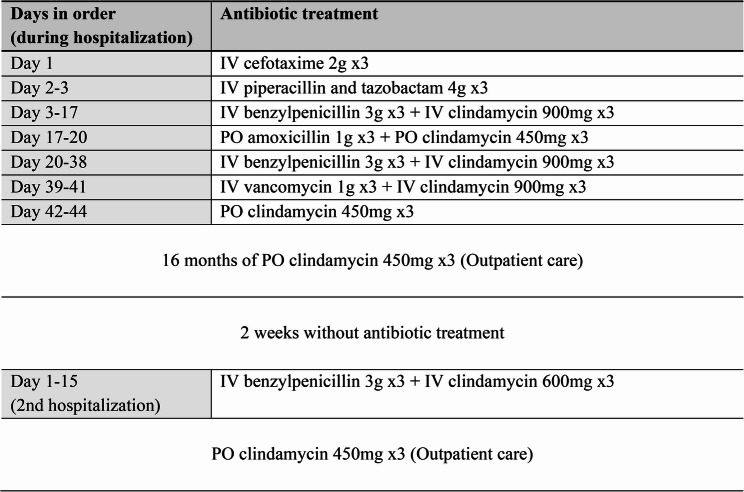
Table of the antibiotics administered in order. Name of given antibiotic treatment in the right column and days in order in the left. On day 44, the patient was discharged from hospital with PO clindamycin 450 mg x3 for 16 months, following 2 weeks without treatment. Subsequently, the patient was again hospitalized for 15 days. Once discharged, the patient was set on the same PO regimen but with lifelong treatment

Subsequent to the findings, the antibiotic regimen was changed to IV benzylpenicillin (3 g three times a day) combined with IV clindamycin (900 mg three times a day). The MIC value for benzylpenicillin was 0.06 mg/L and 0.5 mg/L for clindamycin (Fig. 2.). Consequently, a decrease in CRP from 252 to 36 was seen between day 3 and day 17, along with subsiding pain and the absence of fever or neurological symptoms. Hence, after 17 days of therapy, the IV antibiotics were replaced by oral (PO) clindamycin (450 mg three times a day) and amoxicillin (1 g three times a day).

**Fig. 3:**
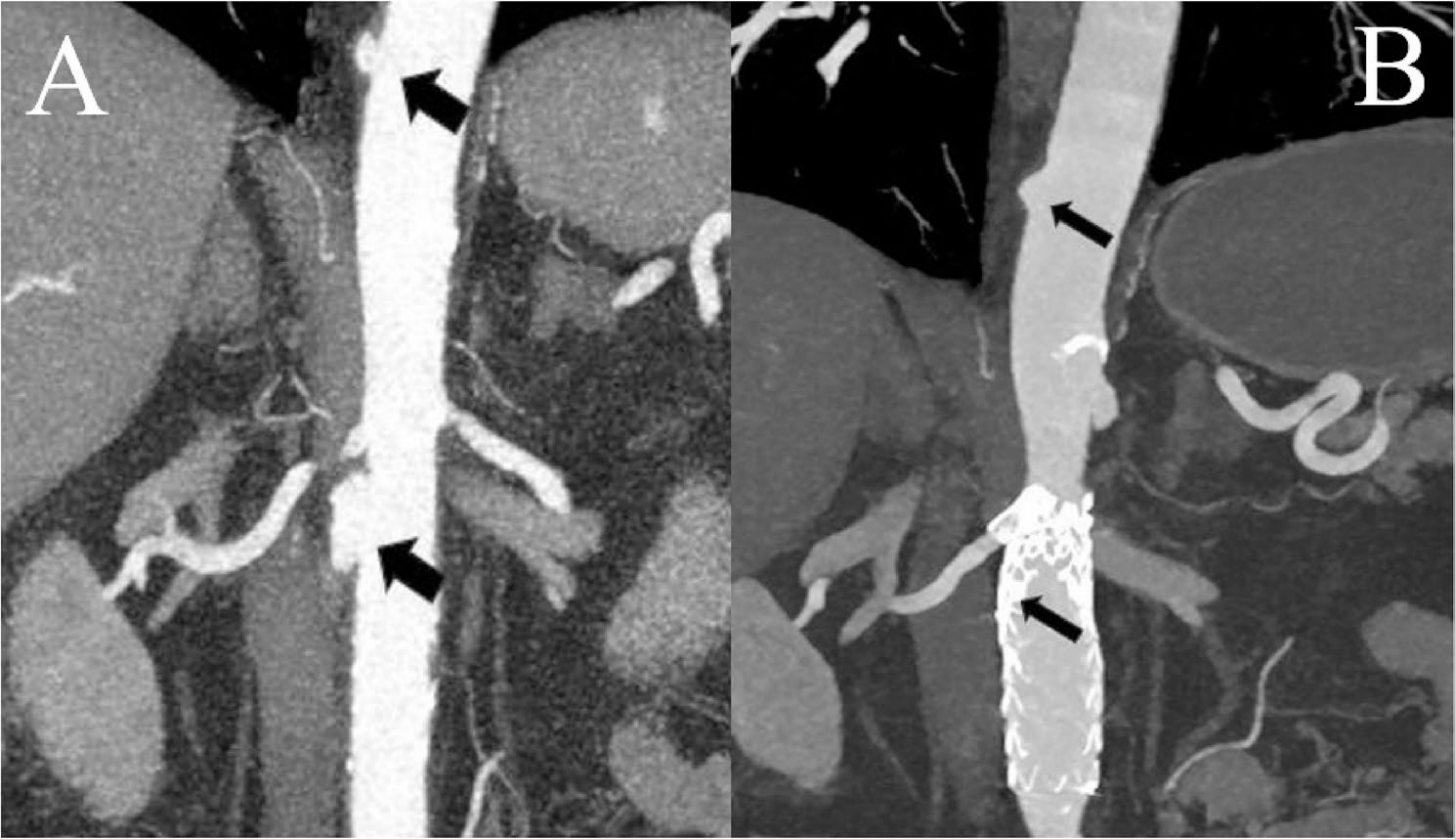
**A** CT abdominal aorta displaying an infrarenal, infected aneurysm and a small ulceration in the distal thoracic aorta (both marked with black arrows). **B** CT abdominal aorta one year postoperatively displaying a stented infrarenal aorta without a visible aneurysm and a minor ulceration in the distal thoracic aorta without inflammatory signs (both marked with black arrows)

**Fig. 4:**
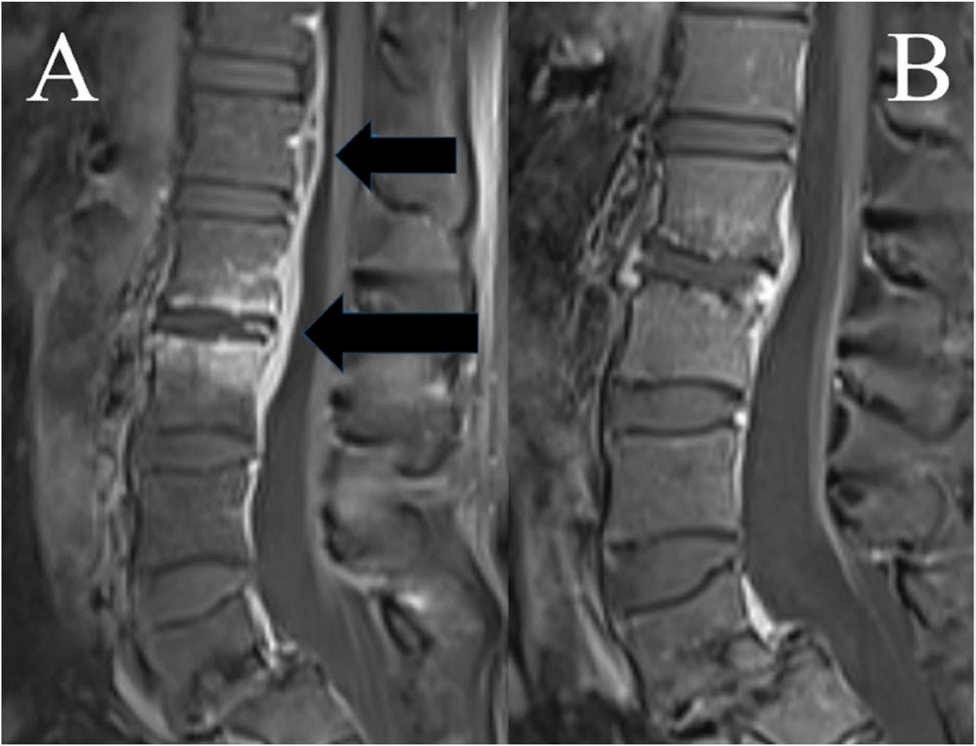
**A** MRI of the lumbar spine during the first hospitalization, showing edema in the L2-L3 intervertebral disc and contrast-enhancement in the endplates of L2-L3 indicating spondylodiscitis. Dorsal to L1, an epidural abscess can be seen (30 × 6 mm). **B** MRI of the lumbar spine 8 months after hospital discharge showing regression of the epidural abscess as well as partial regression of the contrast-enhancement in the paravertebral and prevertebral structures of L2-L3

On day 20, three days after the transition to PO antibiotics, blood cultures were negative but a temperature of 38,3 °C was registered. This motivated additional imaging to evaluate source control. CT revealed an infected (mycotic) infrarenal aneurysm not seen on the previous scan, as well as some new minor aortic ulcerations in the diaphragmatic section of the aorta (Fig. 3, A). In addition, MRI showed spondylodiscitis as well as emerging bilateral psoas abscesses. The epidural phlegmon had spread to the level of thoracic vertebra 12 and an epidural abscess was seen dorsal to L1 (Fig. 4, A). This again prompted IV treatment with benzylpenicillin (3 g three times a day) and clindamycin (900 mg three times a day). An orthopedic surgeon was consulted, proposing conservative management of the spondylodiscitis given the subsiding pain and continuous absence of neurological symptoms.

Considering the rapidly evolving aortic lesions, vascular surgery was consulted. The aneurysm was saccular with an irregular thick wall and a maximum diameter of 34 mm, compared to the adjacent aortic diameter of 20 mm. Open surgery was considered unsuitable due to inherent risks. On day 25, the largest infrarenal aortic lesion was treated with a stent graft to decrease the risk of rupture. The procedure was done under local anesthesia and two tube grafts were inserted just below the right renal artery covering 75 mm of infrarenal aorta, whereas the right renal artery was protected with a separate chimney stent. The minor ulcerations in the distal descending aorta were left for surveillance since covering all lesions would carry a high risk of spinal cord ischemia. The postoperative phase was uneventful.

The two weeks following the procedure, proceeded with decreased pain and reduced inflammatory markers. Neutropenia was noted as a side effect of long-term treatment with beta-lactams, hence, IV benzylpenicillin was replaced by IV vancomycin (1 g three times a day) for the last week of hospitalization (MIC value 2 mg/L for vancomycin). On day 44 the patient was discharged from the hospital with a continuous treatment with PO clindamycin (450 mg three times a day). Due to the correlation between *C. septicum* and malignancy, the patient also underwent colonoscopy that showed nonspecific erosions of the colonic mucosa with negative biopsies. The significance of this in terms of representing the portal of microbial entry was however not determined. On the same note, the patient underwent an investigation for hematological disorders, showing no marks of such malignancy.

During the ensuing year, the patient was kept on clindamycin with monthly outpatient visits showing no signs of clinical deterioration and CRP remained < 1 mg/L. MRI of the lumbar spine 8 months after hospital discharge showed regression of the epidural abscess as well as partial regression of the spondylodiscitis around L2-L3 (Fig. 4, B). Follow-up CT after one year showed that the stented infrarenal aneurysm had disappeared completely and the minor ulcerations in the distal thoracic aorta had decreased in size (Fig. 3, B).

After 16 months on PO clindamycin, the antibiotic treatment was terminated based on radiological signs of regression and the absence of clinical symptoms. However, after 2 weeks without antibiotic therapy, the patient presented at the emergency department again with sudden onset of back pain. During the following two days of hospitalization, CRP went from 8 to 326 mg/L and *C. septicum* was again found in the blood cultures (time-to-detection was 13.9 h in the anaerobic bottle and 104.8 h in the aerobic bottle). MRI revealed progressed spondylodiscitis with epidural phlegmon along L2-L3 and small abscesses in the left psoas muscle. CT also showed discrete signs of graft infection, although not severe enough to motivate new vascular surgery. The patient was set on a regimen of IV benzylpenicillin (3 g three times a day) and IV clindamycin (900 mg three times a day), which successively normalized CRP and reduced the patient’s back pain in 2 weeks. Subsequently, the patient was discharged from the hospital with yearly follow-up imaging of the aorta and lifelong suppressive antibiotic therapy (SAT) with PO clindamycin (450 mg three times a day), deemed as a necessary measure to prevent relapse.

## Discussion and conclusions

### Antibiotic treatment of C. septicum

To date, there is limited in-vivo data on antibiotic treatment of *C. septicum* and its species-specific breakpoints. However, one study from 2018 evaluated the in-vivo efficacy of benzylpenicillin, tetracycline, clindamycin, and vancomycin in murine models of *C. septicum* induced myonecrosis [10]. MIC values were determined for strains from the American Type Culture Collection (ATCC) and clinical isolates collected from patients diagnosed with *C. septicum.* All strains exhibited high sensitivity to benzylpenicillin, tetracycline, and clindamycin (≤ 0.064 µg/mL). In vivo, these three antibiotics also demonstrated full protection against fatal infection when administrated early and in multiple doses. Vancomycin exhibited higher MIC values than other antimicrobials (0.5–1.0 µg/mL) and treatment with vancomycin solely displayed 40% mortality in inoculated mice [10]. The results support the recommendations by the Infectious Disease Society of America (IDSA) suggesting a combination of high-dose intravenous penicillin and clindamycin as first-line treatment of clostridial myonecrosis including cases of *C. septicum* [11]. Added to that, clindamycin could provide a more beneficial protection against alpha-toxin synthesis, arguing for the choice of a treatment combination [12]. This motivated our choice of combining IV benzylpenicillin with IV clindamycin, given that the MIC values were roughly similar to those of the study from 2018. In our case, this combination eventually resulted in a positive therapeutic outcome.

The choices of PO antibiotics were motivated by several factors. First, the selection of clindamycin as monotherapy was based on the above stated effect on toxin synthesis as well as its oral bioavailability and high level of bone penetration, even in the presence of low serum concentrations [13]. Adding to this, the MIC value for clindamycin was low and demonstrated favorable clinical response during the outpatient visits. The decision to continue with lifelong SAT was made due to the swift clinical relapse after quitting the treatment, indicating a high risk of progression of the aortic infection and spondylodiscitis. Of note, the clinical progression following the 3-day course on amoxicillin could be further discussed. The choice of amoxicillin was motivated by its documented antibacterial activity against gram-positive and anaerobic bacteria, similar to that of benzylpenicillin, as well as its high serum levels after oral administration [14–16]. However, the clinical outcome indicated that the shift to PO administration was probably made to early.

### Previous reports

To the best of our knowledge, only three cases of spinal infection caused by *C. septicum* have been reported [6–8], of which one patient presented with epidural abscess [8]. The antibiotic treatment of these patients mainly consisted of IV benzylpenicillin, ampicillin/sulbactam and vancomycin, or either of these in combination. In two of the published cases, surgical intervention was performed as conservative management failed [6, 8]. In none of the mentioned studies, the patients developed infective aortic aneurysms. Although being a rare complication, it is well described in the literature, with high mortality (27–100%) even with antibiotics and surgical intervention [4]. Some reports recommend open surgery rather than endovascular stents, but data is limited [4]. These previous reports did urge our team to do repeated radiology at the time of recurring fever. This revealed rapidly developing aortic aneurysms, although IV antibiotics were chosen based on definitive etiologic diagnosis and in accordance with the IDSA guidelines, in an immunocompetent patient with few symptoms. Of note, except having fever, the patient was clinically unaffected and did not show any other signs of inflammation.

## Conclusions

To our knowledge, this is the first described case of a patient with both musculoskeletal, epidural, and aortic infection caused by *C. septicum*. This case illustrates the necessity to rule out possible aortic infection in patients diagnosed with *C. septicum* bacteremia, even in the setting of clinical improvement and absence of bacteria in follow up blood cultures. It also provides an example of how subtle clinical signs of progression can represent a dramatic development of a clinical scenario, which may demand surgical intervention.

## Data Availability

All data analyzed during this study was achieved through the patient record and is included in this study.

## Abbreviations

ATCC: American type culture collection
BC: Blood culture
CRP: C-reactive protein
CT: Computed tomography
C. septicum: Clostridium septicum
C. perfringens: Clostridium perfringens
IDSA: Infectious diseases society of america
IV: Intravenous
L(n): Lumbar vertebrae (number)
MIC: Minimum inhibitory concentration
MRI: Magnetic resonance imaging
PO: Per os
SAT: Suppressive antibiotic therapy
